# Changes in the severity and subtype of Guillain-Barré syndrome admitted to a specialist Neuromedical ICU over a 25 year period

**DOI:** 10.1007/s00415-016-8380-0

**Published:** 2017-01-16

**Authors:** C. J. Taylor, N. P. Hirsch, D. M. Kullmann, Robin S. Howard

**Affiliations:** Batten/Harris Neuromedical Intensive Care Unit, National Hospital for Neurology and Neurosurgery, Queen Square, London, WC1N 3BG UK

**Keywords:** Guillain-Barré syndrome, Acute idiopathic demyelinating neuropathy, Neurocritical care

## Abstract

We report a retrospective review of 110 patients with acute Guillain-Barré syndrome (GBS) admitted to a specialised intensive care unit (ICU) in a tertiary referral centre over a 25 year period, the start of which coincided with the widespread introduction of plasma exchange (PE) and intravenous immunoglobulin (IVIG). The results were analysed by comparing 52 patients admitted in the first decade (1991–2000; Group 1) with 58 patients admitted between 2001–2014 (Group 2). Patients in both groups were comparable with respect to age and sex, and had a similar incidence and range of ICU complications. They received a comparable range of immunomodulatory treatments including IVIG and PE. However, the delay from presentation to referral to the tertiary ICU was longer in patients in Group 2. They also required mechanical ventilation for a longer duration, and had longer ICU and hospital stays. In Group 2, there was a higher incidence of axonal neuropathy (51%, compared to 24% in Group 1). Despite the longer delay to referral, the prevalence of axonal neuropathy and the duration of ventilation, overall mortality showed a downward trend (Group 1: 13.5%; Group 2: 5.2%). There was no late mortality in either group after step-down to neuro-rehabilitation or following discharge home or to the referring hospital. The rehabilitation outcomes were similar. This data show a shift in the pattern of referral to a tertiary referral ICU between the first and second decades following the wider availability of IVIG and PE for the treatment of GBS. The possible causes and implications of these findings are discussed.

## Introduction

Several randomised controlled trials have established the effectiveness of both plasma exchange (PE) and intravenous immunoglobulin (IVIG) in Guillain-Barré syndrome (GBS). Despite this approximately one third of patients develop respiratory failure requiring tracheal intubation and ventilatory support [1–7]. Many of these patients also require intensive care because of profound bulbar and limb weakness and autonomic instability.

The intensive care management of acute GBS has evolved with the introduction of new modes of ventilation, better techniques of supportive care and the widespread availability of IVIG as a more convenient form of immunomodulatory treatment than PE [8–10]. In the UK, highly specialised neurological intensive care has become easier to access with the development of neuroscience centres, although there is considerable variation in the provision of neurological support for patients admitted to general ICUs. It remains uncertain whether these changes have led to a significant change in the pattern of referral for specialist care or an improvement in the management and outcome of GBS.

In a previous study of 79 patients with acute GBS from the neuromedical ICU (NMICU) at the National Hospital for Neurology and Neurosurgery (NHNN) the overall mortality was 5.1% although 15% remained severely disabled at 6 months and 10% at 1 year [11]. Several other large series of patients treated for GBS in ICUs have subsequently been published. The mortality has varied from 6.5 to 12.2% but there were significant differences in the severity and clinical pattern of the cases seen and treated [12–15]. The majority of patients died from the complications of intensive care and prolonged immobility. Major complications, including pneumonia, sepsis, pulmonary embolism and gastrointestinal bleeding developed in 60% of intubated patients [16]. Approximately, 75% of patients will regain some degree of mobility but slow recovery is well recognised particularly in patients who develop ICU complications, require prolonged ventilation or who have severe axonal loss. In one study [17] the mortality at 1 year was 20% in the group that required mechanical ventilation and recovery was delayed in a sizeable proportion of the survivors.

The long-term outcome of GBS requiring intensive care not only reflects complications and mortality on the ICU. In one study, although the mortality on ICU was 7.7% acute hospital mortality occurring after discharge from ICU was 16.7% [18], underlining the importance of step-down care of patients with GBS. Other reports have also suggested significant late mortality following discharge from ICU [8, 12, 19, 20].

The histological features of GBS support a distinction between acute inflammatory demyelinating polyneuropathy (AIDP) and acute motor axonal polyneuropathy (AMAN) or acute motor and sensory neuropathy (AMSAN), which can be discriminated on nerve conduction studies [21–23]. However, there remains a considerable overlap and the incidence of AIDP may be overestimated if the nerve conduction studies are undertaken early in the course of the disease. The most consistent prognostic features seem to be age, severity of nadir and rapid deterioration to nadir. It is unclear whether the histological type of GBS is relevant to outcome although there remains a clinical view that the prognosis for AMAN or secondary axonal loss in AIDP is worse than isolated AIDP [24, 25].

The extensive and prolonged experience of neurological ICU at The National Hospital for Neurology and Neurosurgery affords a unique opportunity to study long-term trends in the referral patterns and outcome of patients with acute GBS for specialist Neuro-Critical Care support. This retrospective study spans a 25 year period, starting at the time when randomised controlled trial evidence emerged for the effectiveness of immunomodulatory treatment with PE and IVIG.

## Methods

We divided adult patients seen on the specialist neuromedical ICU (Batten Harris unit) at the National Hospital for Neurology and Neurosurgery into two cohorts: those admitted in the years 1991–2000, and those admitted in 2001–2014. The first cohort overlaps with the previous series published from this unit. We excluded cases subsequently found to have mimics of Guillain-Barré syndrome including chronic inflammatory demyelinating polyneuropathy. The primary reason for ICU admission, the presence of overt bulbar and autonomic dysfunction, immunomodulatory treatment, and the need for and duration of mechanical ventilation were described, as was the incidence of tracheostomy if indicated. In addition, patients were classified into ‘early referrals’ if the interval between onset of symptoms and arrival at ICU was <6 days and ‘late referrals’ if after this period.

Nerve conduction studies and electromyography were performed on the ICU although electrical interference from ventilatory and plasmapheresis equipment sometimes restricted the procedure. When possible, limb temperature was maintained at about 32 °C. Sensory and motor conduction studies were typically performed using surface electrodes on the median, ulnar, sural and common peroneal nerves of one side and often the superficial peroneal and posterior tibial nerves as well. F-wave studies were performed on motor nerves, but proximal stimulation at the level of Erb’s point or the spinal column was not carried out on the ICU. For sensory studies, amplitude, distal latency and in most patients, sensory conduction velocities, were recorded. Distal motor latencies, motor conduction velocities and amplitudes of compound action potentials to distal proximal stimulation were also measured. Concentric needle electromyography was performed in general on a proximal and a distal muscle of an upper and lower limb.

The baseline and follow-up electrodiagnostic studies were reviewed and each patient was classified into electrophysiological subtypes based on their initial study as defined by Hadden et al. [22]. We determined whether patients developed low compound muscle action potential (CMAP) amplitudes (<20% lower limit of normal in at least two nerves) or inexcitable nerves (CMAP absent in all nerves or <10% lower limit of normal in one nerve and absent in all other is tested) at any point during their illness. Demyelination refers to patients with the features of AIDP. These patients may later develop secondary axonal loss. Axonal refers to presentation with AMAN or AMSAN.

All ICU complications were documented including hyponatraemia (serum sodium <135 mmol/l) and abnormalities of liver function. Major morbidity was defined as serious infection (ventilator acquired or aspiration pneumonia, sepsis), deep vein thrombosis, pulmonary embolism, severe arrhythmia, haemodynamic instability, gastrointestinal haemorrhage, complications of tracheostomy, pseudomonas colitis due to *clostridium difficile,* ileus with or without bowel perforation and pain.

Outcome measures included length of stay (LOS) in the ICU and the hospital; mortality in the ICU and in the hospital. Functional recovery was extracted from standardised collected assessments of Barthel score at discharge in those patients undergoing rehabilitation at NHNN. Data were not available for those patients undergoing rehabilitation elsewhere.

Values are expressed as mean ± SD or as median (interquartile range). The Fisher exact test and the Kolmogorov–Smirnov test were used to compare across cohorts for categorical and quantitative data, respectively.

## Results

Group 1 (admitted 1991–2000) consisted of 52 patients (mean 5.2 patients/year; 56% male) and Group 2 (2001–2014) included 58 patients (mean 4.5 patients/year; 62% male; Table 1). The mean age in Group 1 was 52 years (range 21–95) and in Group 2 48 years (range 15–82). There were seven deaths in Group 1 (13.5%) and three in Group 2 (5.2%). The decrease in mortality did not reach significance (Fisher exact test: *p* = 0.19). In Group 1, the most common cause of death was overwhelming sepsis (four patients); other causes were blocked tracheostomy, cardiogenic shock with bowel ischaemia, and hypoxic-ischaemic brain injury. In Group 2, two patients died of sepsis-related shock and one of cardiogenic shock.

**Table 1:** Demographic characteristics of the two patient groups

	Group 1 1.1991–12.2000 (120 months)	Group 2 1.2001–12.2014 (156 months)	
*n*	52	58	
M:F	29:23	36:22	
Age (years) mean (±SD) (median) (range)	52 (±19) (48) (21–95)	48(±18) (48) (15–82)	NS
Time to referral [after onset (days)] <3	35 (76%)	23 (40%)	
4–5	5	6	
>6	6 (12%)	24 (41%)	*p* = 0.0005
Unknown	6	5	
NMICU stay (days) mean (±SD) (median) (range)	40 (±38) (29) (3–223)	59 (±54) (44) (8–263)	*p* = 0.051
Hospital LOS (days) mean (±SD) (median) (range)	82 (±104) (56) (6–643)	108 (±83) (103) (11–424)	*p* = 0.047
Intubated	51	58	
Tracheostomy	49	58	
Time to tracheostomy (days)	8 (±7) (1–39)	6 (±5) (1–33)	
Duration of Ventilation Mean (± SD) (Median) (Range)	44 (±63) (26) (10–315)	59 (±47) (54) (12–253)	*p* = 0.001
*Neurophysiology*			
Demyelinating	38 (76%)	28 (48%)	
Mean age (years) (±SD) (median) (range)	54 (±18) (54) (26–88)	47(±18) (48) (17–82)	
Duration of ventilation Mean (±SD) (median) (range)	37 (±38) (20) (10–223)	45(±28) (45) (12–118)	
Hospital LOS mean (±SD) (range)	82 (±111) (4–643)	86 (±41) (11–142)	
Died	4	2	
Axonal	12 (24%)	30 (51%)	
Mean age (years) (±SD) (median) (range)	44 (±20) (38) (20–77)	49(±19) (52) (18 = 78)	
Duration of ventilation mean (±SD) (median) (range)	48 (±38) (40) (6–115)	74(±58) (60) (13–253)	
Hospital LOS mean (±SD) (range)	88(±85) (6–327)	130 (±102) (8–260)	
Mortality			
Died	3	1	
Overall Treatment			
Only Ig	40 (multiple: 5)	51 (multiple: 15)	
Only PE	17	12	
Both	12	12	
Rehabilitation			
n	30	32	
Made gains on Barthel	29	30	
Mean gain on Barthel	6.9	9.7	
Barthel 18–20 on discharge	17/21 (81%)	27/32 (84%)	
Discharged			
Home	33	35	
Another hospital	7	9	
ICU	2	1	
Long term care	1	1	
Unknown	2		

Six patients in Group 1 were referred late, as opposed to 24 patients in Group 2 (Fisher exact test: *p* = 0.0005). The mean length of ICU stay in Group 1 was 40 days (median 29) and in Group 2, 59 days (median 44). The distribution of length of stay was, however, skewed with 12 patients staying longer than 50 days in Group 1 and 26 in Group 2. The longer stay for Group 2 was borderline-significant with a Kolmogorov–Smirnov test (*p* = 0.051). The mean hospital length of stay in Group 1 was 82 days (median 56) and in Group 2, 108 days (median 103, *p* = 0.047) with 11 patients in Group 1 staying longer than 100 days compared to 26 in Group 2.

In Group 1, three patients did not require tracheostomy and were rapidly extubated. All the patients in Group 2 required tracheostomy.

The mean duration of ventilation was available for 37 patients in Group 1 and 42 patients in Group 2. In Group 1 it was 44 days (median 26 days; range 10–315) and in Group 2, 59 days (median 54; range 12–253, *p* = 0.001). All the patients in both groups were weaned from ventilatory support before their transfer to step-down care, rehabilitation or the referring hospital.

Nerve conduction studies showed that in Group 1, 38 (76%) cases were primarily demyelinating and 12 (24%) axonal (2 unknown) whilst in Group 2, 28 (48%) were primarily demyelinating and 30 (52%) axonal. Excluding cases where the subtype was not established, the difference in prevalence of axonal neuropathy was significant (*p* = 0.006) (Fisher exact test).

We asked if the subtype of neuropathy correlated with age or duration of ventilation. In Group 1, the mean ages for demyelinating and axonal neuropathy subtypes were 54 and 44; the mean duration of ventilation was 37 vs. 48 days; and the mean length of hospital stay was 82 vs 88 days. In Group 2, the mean age for demyelinating and axonal subtypes were 47 and 49, respectively; the mean duration of ventilation was 45 vs. 74 days; and the mean length of hospital stay was 86 vs 130 days. When Groups 1 and 2 were pooled, the duration of ventilation was significantly longer for patients with axonal neuropathy (Kolmogorov–Smirov test, *p* = 0.002).

There was no difference between the two cohorts with regard to rehabilitation and gains made on Barthel score. There was no late mortality between discharge from ICU and from NHNN either home or to a referring hospital. None of the patients required continuing non-invasive ventilatory support. The place of discharge is shown in Table 1.

The antecedent precipitating factors are summarised in Table 2. The incidence and nature of autonomic and systemic complications during ICU care was similar with a high frequency of pulse and blood pressure instability, bowel disturbance, ventilator acquired pneumonia, hyponatraemia, sepsis and tracheostomy complications (see Table 3).

**Table 2:** Antecedent factors precipitating acute Guillain-Barré syndrome

	Group 1 1.1991–12.2000	Group 2 1.2001–12.2014
Campylobacter	13	8
Diarrhoea (culture negative)	6	6
Respiratory tract infection	12	15
CMV	2	1
Mycoplasma	1	2
Others	5	2

**Table 3:** ITU complications

	1.1991–12.2000	1.2001–12.2014
Generalised autonomic instability	43	38
Haemodynamic instability	31	31
Bowel disturbance (ileus, severe constipation)	8	11
Percutaneous endoscopic gastrostomy	2	2
Abnormal liver function	9	12
Hyponatraemia	7	8
Ventilator associated pneumonia/aspiration pneumonia	28	20
Sepsis	7	8
Tracheostomy complications	6	7
Uncontrolled severe pain	11	10
Urinary disturbance	12	9
Acute kidney injury	2	3

## Discussion

This retrospective study looked at referral patterns of patients with severe GBS to a specialised neurological ICU over a 25-year period. It has shown a striking change in practice. Patients across the two described cohorts were of a similar age and sex, had a similar incidence and range of ICU complications and a comparable range of immunomodulation treatment. However, patients admitted in the later cohort, between January 2001 and December 2014, were referred later in their illness, received mechanical ventilation for a longer time, and required longer ICU and hospital stays. In the latter group, there was a much higher incidence of axonal neuropathy, possibly explaining the increased duration of ventilation and length of stay. There was also a non-significant trend towards lower mortality despite a longer duration of mechanical ventilation.

The data suggest that patients are increasingly referred later in the course of the disease, when more profoundly impaired as a result of respiratory muscle, bulbar and limb weakness and dependency and with more severe axonal forms of the condition. Despite the referral of more severely impaired patients the ICU mortality was no greater, there was no step-down mortality, and the discharge outcome was unchanged.

We speculate that in the earlier cohort patients were transferred earlier in the course of the condition to a limited number of specialist units able to provide PE. The increasing use of IVIG as a first line treatment has meant that most patients with Guillain-Barré syndrome are managed in the ICU of the admitting hospital. Clinical experience suggests that this change in practice may extend to the management of patients with severe acute Guillain-Barré syndrome requiring ventilatory support. This means that patients are transferred to specialist units only if their condition fails to improve, if they continue to require prolonged mechanical ventilation, or if they develop severe complications of the primary condition. To some extent, this change in practice may reflect the limited provision of specialist Neurocritical care in the UK.

The study is necessarily limited because of its retrospective nature. The analysis is based on the review of medical records originally completed by multiple different observers although it should be emphasised that three of the authors (NPH, RSH, DMK) were involved in the management of all of the patients. The sample was inevitably selected by the threshold for referring patients with varying severity and the duration of disease from primary to secondary or tertiary specialist units. The delays from symptom onset to hospital admission, from hospital admission to ICU transfer and from symptom onset to the provision of IVIG were not always available, similarly, when administered, the indication for first treatment with IVIG in the referring hospital was not always clear. The Barthel score at discharge was not available for all patients in Group 1. The timing of nerve conduction studies in relation to onset and the protocols used inevitably varied considerably over the 24 years of the study, particularly as many were undertaken in referring hospitals. We recognize that the variable timing of neurophysiology studies will have meant that some patients with inexcitable nerves may have had an initial demyelinating neuropathy. Prospective studies show that it can be difficult to classify the underlying neuropathy in 10–15% of patients. In this study, we considered all patients with severe axonal loss at the time of nerve conduction studies to have had a primary axonal neuropathy but it is likely this would lead to an over-representation of the axonal form in our results. Despite these limitations, this study provides unique information about changes in the pattern of referral to specialist ICU for GBS in the UK over the past 25 years.

Delay in transferring patients with acute GBS to specialist ICU may occur for several reasons, including: wider provision of primary general intensive care across the UK, meaning that acutely ill patients are admitted, intubated and ventilated sooner and appropriate treatment is commenced with the minimum delay; better acute management and ICU care of Guillain-Barré syndrome in primary ICUs;, improved neurological input to ICUs in general hospitals; wider availability of IVIG and wider access to neuro-rehabilitation facilities. More specialised ICU beds also allow patients to be treated with high level medical and nursing support for longer. The data suggest an increased threshold for transferring patients to highly specialised units; this may be because of improved supportive care in primary ICU and the more rapid and easier availability of immunomodulatory treatment.

The optimal management of severe AIDP and AMAN remains uncertain and, in particular, the mechanisms and management of patients who deteriorate after initial improvement or continue to deteriorate after receiving first-line immunomodulatory treatment is not clear. It is perhaps these patients who should be transferred to specialised units to allow a trial of repeated IVIG treatment or to consider the role of combined plasma exchange followed by IVIG.

These findings raise a number of issues and concerns. The change in practice in the UK suggested by this series, is primarily likely to reflect increasing ICU provision, better management guidelines and a wider availability of neurological support. All these factors have probably led to more patients remaining for longer periods in primary ICUs without the need for transfer to specialist units. Indeed the recent mortality data for ICU care of acute Guillain-Barré syndrome in the UK, has been favourable [18]. However, the acute hospital mortality data in other series suggests an under-appreciation of the risks in step-down care. The absence of any late mortality in this series, following discharge from ICU, may reflect the availability of continuing neurological support in a specialist hospital. It is clear that future studies must seek to define more clearly the indications, guidelines and timing of transfer from general ICU to specialist neuroscience intensive care.

There is a debate about the place of highly specialised neurological intensive care, and it is uncertain if their primary role should lie in managing patients with common presentations of acute neurological disorders or if the scarce resources should be focused on the specialised care of tertiary referrals of the most complex and difficult management problems, which often demand extensive time and resource input to achieve the best outcomes. If this is the case it will be impossible to prove such units improve the mortality and morbidity rate of neurological disorders. However, they will have an important role as centres of last resort and in teaching, research and establishing guidelines of care.

In conclusion, the data presented in this paper argues that the overall outcome of patients referred to a specialised ICU with Guillain-Barré syndrome has, at least, remained stable despite referral of more severely affected patients. Review of the recent literature indicates that the failure to refer some severely affected patients to specialised units might contribute to the high acute hospital mortality for the condition in the UK. This data argue strongly for an improvement in the provision of Neurocritical care and earlier referral of patients with severe acute Guillain-Barré syndrome in whom the need for prolonged mechanical ventilation is anticipated.
